# *illuminaio*: An open source IDAT parsing tool for Illumina microarrays

**DOI:** 10.12688/f1000research.2-264.v1

**Published:** 2013-12-04

**Authors:** Mike L Smith, Keith A. Baggerly, Henrik Bengtsson, Matthew E. Ritchie, Kasper D. Hansen

**Affiliations:** 1 CRUK Cambridge Institute, Li Ka Shing Centre, The University of Cambridge, Cambridge, CB2 0RE, UK; 2 Department of Bioinformatics and Computational Biology, The University of Texas MD Anderson Cancer Center, Houston, TX 77030, USA; 3 Department of Epidemiology and Biostatistics, University of California, San Francisco, CA 94107, USA; 4 Molecular Medicine Division, The Walter and Eliza Hall Institute of Medical Research, Parkville, Victoria 3052, Australia; 5 Department of Mathematics and Statistics, The University of Melbourne, Parkville, Victoria 3052, Australia; 6 McKusick-Nathans Institute of Genetic Medicine, Johns Hopkins School of Medicine, Baltimore, MD 21205, USA; 7 Department of Biostatistics, Johns Hopkins Bloomberg School of Public Health, Baltimore, MD 21205, USA

## Abstract

The IDAT file format is used to store BeadArray data from the myriad of genomewide profiling platforms on offer from Illumina Inc. This proprietary format is output directly from the scanner and stores summary intensities for each probe-type on an array in a compact manner. A lack of open source tools to process IDAT files has hampered their uptake by the research community beyond the standard step of using the vendor’s software to extract the data they contain in a human readable text format. To fill this void, we have developed the illuminaio package that parses IDAT files from any BeadArray platform, including the decryption of files from Illumina’s gene expression arrays. illuminaio provides the first open-source package for this task, and will promote wider uptake of the IDAT format as a standard for sharing Illumina BeadArray data in public databases, in the same way that the CEL file serves as the standard for the Affymetrix platform.

## Introduction

The DNA microarray field is dominated by the three manufacturers: Affymetrix, Illumina and Agilent. While the basic premise behind their competing products is the same (i.e. the measurement of hybridisation between sample and immobilised probes on arrays via fluorescence), the formats in which these data are presented to end users are quite different, with each manufacturer electing to use their own proprietary format. The most ubiquitous of these is the CEL file, which has been accepted as a standard format for publishing the raw data generated on the Affymetrix platform. A search of the Gene Expression Omnibus (GEO, http://www.ncbi.nlm.nih.gov/geo/) database finds over 90% of submissions of Affymetrix data include one or more CEL files as supplementary material. The format itself is well documented by the manufacturer, who also provides an open-source software development kit (SDK). As a result, in addition to Affymetrix’s own software suite, a large number of CEL parsing tools exist, including a parser implemented based on the file format documentation: *affyio* ^1^ and a parser based on the SDK: *affxparser* ^2^.

**Figure 1.**
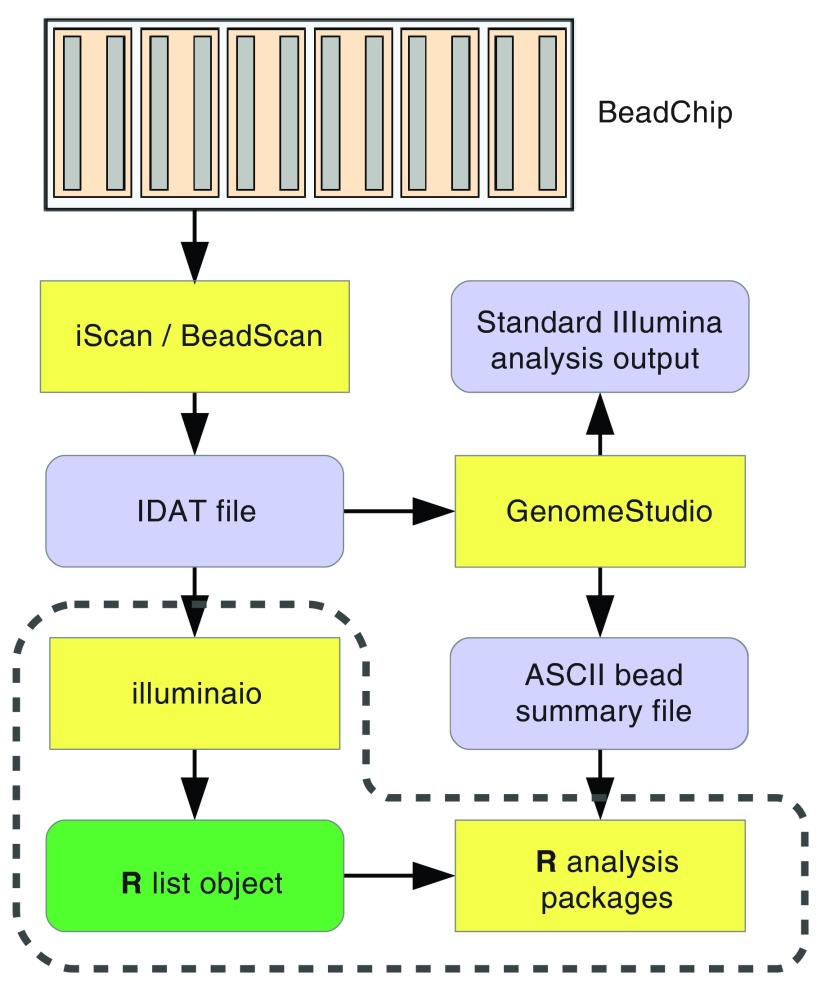
A typical BeadArray analysis workflow. Scanning of BeadChips is performed using the iScan or BeadScan control software, producing IDAT files. Currently, these are read by GenomeStudio where the user has the choice of performing an analysis using that software, or outputting the intensities to a text file for processing by alternative means. *illuminaio* removes this reliance on GenomeStudio. The intention is for analysis packages to incorporate its routines, effectively merging the dashed region and simplifying the analysis process for end users.

The same is not true of the primary IDAT format from Illumina, with only 1.5% (49 out of 3208) of the submissions in GEO that use Illumina BeadArrays including IDAT files as supplementary material. Given that IDATs are the standard file type generated during BeadArray processing, it seems reasonable to assume that the relative dearth of IDAT files in the public domain is due to the lack of widespread support for the format. The development of alternative parsing tools has proven more challenging for IDATs for a number of reasons. The foremost amongst these is a lack of public documentation, leaving tool developers to determine the file structure themselves. A further hurdle has been the encryption of IDAT files generated from expression chips. These barriers initially left researchers reliant on the output from Illumina’s *GenomeStudio* software to convert the data into a more convenient format. Existing open source tools, particularly those that focus on gene expression analysis such as *beadarray* ^3^, *lumi* ^4^ and *limma* ^5^, all require that the IDAT files have been processed using *GenomeStudio* to generate a plain-text ASCII file before any analysis can take place ( Figure 1). The *GenePattern* ^6^ software suite includes support for reading expression IDAT files, although it is limited to extracting only a subset of the array information. *GenomeStudio* output also omits various information that is available from the IDAT, such as control probe intensities (for SNP and methylation platforms), so-called out-of-band probes (methylation 450k) ^7^ and meta information including software versions and scan date (all platforms).

Here we introduce the Bioconductor ^8^ package *illuminaio* that can handle IDAT files from any Illumina BeadArray platform, providing a simple unified interface to various low-level data extraction routines.

## Data format

The IDAT file format varies depending upon the array platform ( Table 1). IDATs generated during the scanning of genotyping and methylation BeadArrays are binary files (one for each of the red and green channels). The bulk of each file is comprised of four fields: the ID of each bead-type on the array, the mean and standard deviation of their intensities, and the number of beads of each type. Additionally, metadata including the date the array was scanned, specific software versions used and the type of BeadChip are also included. Once the structure of the file is understood these binary values can be read directly.

**Table 1.** Summarising the file formats for various BeadArray platforms.

Array type	File format	No. data fields
SNP genotyping	Binary	4
Methylation	Binary	4
Gene expression	Encrypted XML	10

On the other hand, gene expression IDAT files are produced as encrypted XML files. Once decrypted the majority of the data are found as ten Base64 encoded strings. These ten fields include the ID, mean and standard deviation values as found in genotyping IDATs, as well as median and trimmed-mean intensity values, the mean and standard deviation of local background intensities, and the number of beads both before and after outliers have been excluded.

Each array type is also associated with a manifest file (with file extension BPM or BGX) that provides details of probe sequences, intended genomic targets and whether it is a control probe or not, information that is necessary to correctly interpret the data.

## Implementation

*illuminaio* is an R package ^9^. The reading of IDAT files is achieved using the **readIDAT** function. This routine is able to determine the type of IDAT file that has been passed and calls the appropriate code to read the file and return the data as a R list object ( Figure 1). This not only contains intensity data, but also the meta information such as scan date that are not routinely extracted and can be useful for detecting batch effects ^10^.

Decryption of expression IDATs is performed using the open-source DES decryption routine available in *Gnulib* ^11^. There is no official documentation of this file format, but *illuminaio* includes a document describing our findings in detail. Source code for the appropriate routines has been adapted and included in *illuminaio*, removing any requirement for specific external libraries to be installed on a user’s computer. Thus the package can be built and run on all three major operating systems (Linux, Windows and Mac).

The *illuminaio* package also supports the parsing of non-encrypted IDAT files compressed by gzip and the reading of manifest files describing the array design ( **readBGX** and **readBPM**).

The summarised intensity values obtained by *illuminaio* are nearly identical to those reported using *GenomeStudio*. Small discrepancies related to rounding performed by *GenomeStudio* are observed. The package vignette contains a detailed comparison. The time taken to read an IDAT depends on the platform, with encrypted expression arrays taking around 1 second per file (for 50,000 probes), and methylation and SNP platforms between 1 to 6 seconds depending on the chip density (which can range between a few hundred thousand and several million probes).

## Discussion

The availability of an open-source IDAT reader through *illuminaio* that can read files from any of Illumina’s BeadArray technologies will promote greater use of the IDAT file as a primary data format in the analysis and sharing of results from BeadArray based profiling studies. The *illuminaio* package is intended for use by developers to efficiently extract the content of both IDAT and bead-manifest files, thereby expanding the possibilities for conducting reproducible research with these data.

One exception to the dearth of IDAT files noted in the introduction is the The Cancer Genome Atlas (TCGA, http://cancergenome.nih.gov/). IDAT files from Illumina methylation and genotyping arrays are available in large numbers as Tier 1 data from the TCGA website ( https://tcga-data.nci.nih.gov/tcga/). Of particular interest is the Illumina 450k methylation array, for which Triche *et al.* ^7^ has shown improvements in background correction by using out-of-band probes, information that is only available through IDAT files and not the GenomeStudio output. For this work Triche *et al.* used *illuminaio* to access the out-of-band probes, which shows the advantage of having access to low-level data.

*illuminaio* is currently used in the *minfi* ^12^, *methylumi* ^13^ and *crlmm* ^14, 15^ packages for importing IDAT files from the Infinium methylation and genotyping platforms respectively, demonstrating its utility.

## Software availability

*illuminaio* is an R package available from the Bioconductor project ( http://www.bioconductor.org) and from 10.5281/zenodo.7588.

## References

[1] Bolstad BM: affyio: Tools for parsing Affymetrix data files. R package version 1.30.0. Reference Source

[2] Bengtsson H Bullard J Hansen KD: affxparser: Affymetrix File Parsing SDK, R package version 1.34.0.2013 Reference Source

[3] Dunning MJ Smith ML Ritchie ME: beadarray: R classes and methods for Illumina bead-based data. *Bioinformatics.* 2007;23(16):2183–2184 10.1093/bioinformatics/btm311 17586828

[4] Du P Kibbe WA Lin SM: lumi: a pipeline for processing Illumina microarray. *Bioinformatics.* 2008;24(13):1547–1548 10.1093/bioinformatics/btn224 18467348

[5] Smyth GK: Limma: linear models for microarray data. In R. Gentleman, V. Carey, S. Dut, R. Irizarry, and W. Huber, editors, *Bioinformatics and Computational Biology Solutions Using R and Bioconductor*,2005;397–420 Springer, New York. Reference Source

[6] Reich M Liefeld T Gould J: GenePattern 2.0. *Nat Genet.* 2006;38(5):500–501 10.1038/ng0506-500 16642009

[7] Triche TJ Jr Weisenberger DJ Van Den Berg D: Low-level processing of Illumina Infinium DNA Methylation BeadArrays. *Nucleic Acids Res.* 2013;41(7):e90 10.1093/nar/gkt090 23476028 PMC3627582

[8] Gentleman RC Carey VJ Bates DM: Bioconductor: open software development for computational biology and bioinformatics. *Genome Biol.* 2004;5(10):R80 10.1186/gb-2004-5-10-r80 15461798 PMC545600

[9] R Core Team. R: A Language and Environment for Statistical Computing. R Foundation for Statistical Computing, Vienna, Austria,2013 Reference Source

[10] Leek JT Scharpf RB Bravo HC: Tackling the widespread and critical impact of batch effects in high-throughput data. *Nat Rev Genet.* 2010;11(10):733–739 10.1038/nrg2825 20838408 PMC3880143

[11] GNU Project. *Gnulib - The GNU Portability Library* ,2013 Reference Source

[12] Hansen KD Aryee M: minfi: Analyze Illumina’s 450k methylation arrays,2013 R package version 1.8.3. Reference Source

[13] Davis S Du P Bilke S: methylumi: Handle Illumina methylation data, R package version 2.8.0.2013 Reference Source

[14] Ritchie ME Carvalho BS Hetrick KN: R/Bioconductor software for Illumina’s Infinium whole-genome genotyping BeadChips. *Bioinformatics.* 2009;25(19):2621–2623 10.1093/bioinformatics/btp470 19661241 PMC2752620

[15] Scharpf RB Irizarry RA Ritchie ME: Using the R Package crlmm for Genotyping and Copy Number Estimation. *J Stat Softw.* 2011;40(12):1–32 22523482 PMC3329223

