# Supplementary material for: illuminaio: An open source IDAT parsing tool for Illumina microarrays
Source: F1000Res. 2013 Dec 4;2:264. [Version 1] doi: 10.12688/f1000research.2-264.v1 (PMC3968891; doi:10.12688/f1000research.2-264.v1)
Supplement: Peer review history

# Peer review: Referee response for version 1

**Published:** 2014-01-29

**Authors:** Tiffany Morris

**Affiliations:** 1 UCL Cancer Institute, University College London, London, UK

This manuscript written by Smith *et al.* presents an R package to process IDAT files, the file format of Illumina BeadArrays. The package *illuminaio* fills an important gap in data processing by enabling the analysis of Illumina data without Illumina software. The gap is illustrated when the authors cite the disparity between the availability of CEL files (Affymetrix file format) and IDAT files in public data repositories. In addition to eliminating the need to use Illumina software, *illuminaio* extracts additional information from the IDAT files allowing improved quality control analyses and additional data correction algorithms. Figure 1 of the manuscript illustrates the simplified analysis pipeline enabled by *illuminaio*.

 

The authors briefly describe the IDAT file format that was not publically documented before *illuminaio*. Figure 2 of the manuscript summarises the details of the file format for each of the BeadArray platforms. As such this is not just the development of a new tool, as the authors have provided thorough documentation for a widely used platform. In addition, the vignette accompanying *illuminaio *clearly explains the packages methods and how to use them.

 

This package, *illuminaio,* has already led to the development of many new analysis packages that utilise IDAT files and the additional information they provide. In turn these packages are leading to the increased exchange and availability of the raw IDAT files in collaborations and data repositories.

I have read this submission. I believe that I have an appropriate level of expertise to confirm that it is of an acceptable scientific standard.

# Response

**Published:** 2014-02-06

**Authors:** Kasper Hansen

**Affiliations:** khansen@jhsph.edu, USA

Dr. Morris,

Thank you for your positive review. We are happy that you find our tool useful.

Best,

Kasper D. Hansen

# Peer review: Referee response for version 1

**Published:** 2014-01-27

**Authors:** Jan Oosting

**Affiliations:** 1 Department of Pathology, Leiden University Medical Center, Leiden, Netherlands

The article describes illuminaio, an R package to process the raw data files produced by the Illumina scanning software. This tool is valuable, because it enables researchers to use a completely open analysis workflow, without having to use a closed source, blackbox, analysis step.

I have tried the package on IDAT files from several chiptypes, and only files from the beginning of 2005 scanned with beadscan 2.2 were rejected. Files scanned with beadscan 2.3 (Nov 2005) and onwards were processed properly.

I have read this submission. I believe that I have an appropriate level of expertise to confirm that it is of an acceptable scientific standard.

# Response

**Published:** 2014-02-06

**Authors:** Kasper Hansen

**Affiliations:** khansen@jhsph.edu, USA

Dr. Oosting,

Thank you for your review.  I am especially happy that you have taken the effort to apply illuminaio on output from older versions of beadscan.

We have contacted Dr. Oosting offline and have received a number of files from old scans. We have started implementing support for these older files, and initial results are promising. We expect to release a new version of illuminaio shortly, which will read these files.

Best,

Kasper D. Hansen
